# Challenges in the Use of Compact Disc-Based Centrifugal Microfluidics for Healthcare Diagnostics at the Extreme Point of Care

**DOI:** 10.3390/mi7040052

**Published:** 2016-03-24

**Authors:** Jordon Gilmore, Monsur Islam, Rodrigo Martinez-Duarte

**Affiliations:** Mechanical Engineering Department, Clemson University, Clemson, SC 29634, USA; jagilmo@clemson.edu (J.G.); monsuri@g.clemson.edu (M.I.)

**Keywords:** centrifugal, microfluidics, point of care, diagnostic, lab-on-disk

## Abstract

Since its inception, Compact Disc (CD)-based centrifugal microfluidic technology has drawn a great deal of interest within research communities due to its potential use in biomedical applications. The technology has been referred to by different names, including compact-disc microfluidics, lab-on-a-disk, lab-on-a-CD and bio-disk. This paper critically reviews the state-of-the-art in CD-based centrifugal microfluidics devices and attempts to identify the challenges that, if solved, would enable their use in the extreme point of care. Sample actuation, manufacturing, reagent storage and implementation, target multiplexing, bio-particle detection, required hardware and system disposal, and sustainability are the topics of focus.

## 1. Introduction

Recent developments in the area of Point of Care (POC) diagnostics and therapeutics have led many researchers, clinicians, policy makers, and lay people to explore the potential for these technological developments to bring relief to patients in the poorest, most remote areas of the globe. These areas of the world and clinical situations are referred to in this work as the extreme POC. Specific engineering challenges present themselves at this interface. For the purpose of this work and future conversations, the authors are defining the extreme POC as healthcare environments without reliable electricity, clean water, sufficient healthcare or logistics infrastructure, sufficiently trained healthcare work force, monetary resources for sustainable health solutions, or combinations of these issues. Hence, an ideal device for extreme POC is one that is inexpensive in absolute terms and offers reproducible, accurate, easy-to-read and unambiguous results. The conditions must be satisfied while not depending on reliable electricity, controlled temperatures, clean water or medically-trained staff to be operated and/or distributed. In general, any diagnostic developed for the extreme POC should take into account the ASSURED criteria as defined by the World Health Organization [1]; where A stands for affordable, S for sensitive, S for specific, U for user-friendly, R for robust and rapid, E for equipment-free or minimal equipment needed , and D for deliverable to those in need.

While a daunting task, some progress has been made. For example, in the area of microfluidics, paper-based systems have proven to be inexpensive, reliable, versatile, and easily disposed of. One of the most successful applications of paper based microfluidics devices is blood glucose meter [2,3,4] and test strips for diabetic patients [5]. Vella *et al*., presented a paper based microfluidics device to measure two major enzymatic markers of liver function and total serum protein from blood extracted from a finger-stick [6]. Enzyme linked immunosorbent assay (ELISA) has been also realized in several paper based microfluidic devices [7,8,9]. However, these paper microfluidic systems fall short in a number of key areas, namely robustness, specificity and sensitivity. Extreme testing conditions affect the performance of these devices, since challenging conditions can have a negative effect on the reagents used in these devices. The migration speed of the liquid and recognition among molecules in these systems depends on the temperature and the humidity of the surroundings, which leads to varying results in different conditions [10]. The detection of low concentration components is difficult, due to poor hydrophobic barriers which also hinder sample retention and can lead to sample interference [11,12]. In the case of these conditions, a more substantial analysis platform is required for effective patient care. These shortcomings suggest the need for a more sophisticated microfluidic based system still able to accommodate the design constraints presented at the extreme POC.

CD-based centrifugal microfluidics has been an active area of research since the 1990s and is now a technology that is mature enough for commercial clinical applications [13,14]. A number of traditional point-of-care applications have also been reported by different research groups [15,16,17]. Recently, it has emerged as a technology with potential applications at the extreme POC, bridging the gap between over-simplified paper microfluidic systems and over-complex expensive laboratory-based microfluidic systems. As stated before, the extreme POC presents a number of significant challenges and CD-based centrifugal microfluidics may offer solutions to detection of diseases such as malaria, tuberculosis, acquired immunodeficiency syndrome (AIDS), diabetes, and neglected infectious diseases such as Chagas disease and leishmaniasis, in the poorest regions of the world.

Several excellent reviews of CD-based centrifugal microfluidics and microfluidics for diagnosis have been published to date [18,19,20,21,22]. The state-of-the-art has been comprehensively covered by these authors. The aim of the work presented here is not to repeat such efforts but to highlight the potential for application of CD-based centrifugal microfluidic technologies in the extreme POC. We first focus on the current state of the art in CD microfluidics with respect to extreme POC design constraints and applications. The authors then aim at identifying areas of opportunity that may lead to solving some of the grand challenges of the extreme POC through CD microfluidics.

Current CD microfluidic developments focus on fluid actuation, fluid mixing, particle separation, and bio-particle analysis based on manipulation of centrifugal force-based actuation systems, intricate micro-scale channel frameworks, and combination of biologically active reagents and detergents. These developments can be seen as advantages of CD microfluidics over other methods of mixing and filtration. This work categorically addresses these advantages with respect to the extreme POC. Sample actuation, device manufacturing, reagent storage and implementation, target multiplexing, bio-particle detection, and system disposal and sustainability will be covered in the sections below.

## 2. Fluid Actuation

The movement of fluid through microscale channels by centripetal force has been studied and developed by researchers since the late 1960s [23]. Centrifugation as a method of actuation has been explored as an alternative to traditional pumps because its lack of need for extraneous equipment (*i.e*., syringes, tubing and syringe pumps), the ability to contain an entire assay on a single disc, and the ability to actuate many fluid types due to its independence of movement from physiochemical properties (*i.e*., pH or ionic strength) [19,21]. A number of functions have been accomplished using centrifugation as an actuation method and include fluid mixing, valving, volume definition (metering), and multidirectional actuation.

### 2.1. Fluid Mixing

The mixing of fluid, be it multi-sample mixing, sample-reagent mixing, or fluid agitation are all critical components to the application of centrifugal microfluidic technology. One of the early barriers of this technology was that mixing was diffusion limited due to the viscosity-dominated flow characteristics (laminar flow) [24]. Mixing two or more fluids in a microfluidic channel or reservoir was limited by the concentration of each fluid and inversely proportional to the rotation speed of the disc. One of the solutions to this issue is the introduction of magnetic beads driven throughout the microchannel by fixed magnets as the CD is rotated at high speeds. The magnetic field generated by the spinning of the CD causes the beads to agitate in a chaotic manner. This agitation causes a rapid mixing of the surrounding fluid [25,26,27,28].

**Figure 1:**
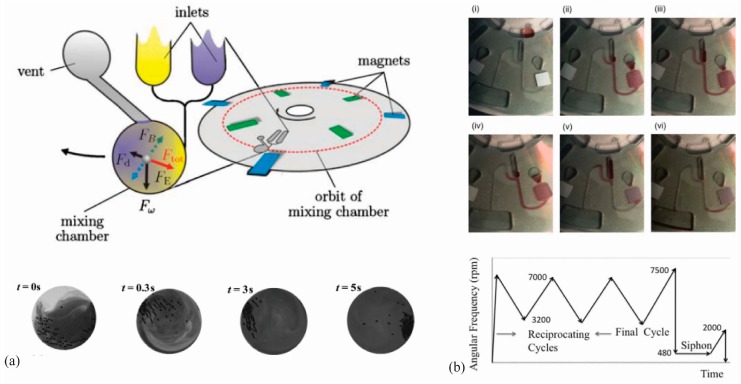
(**a**) Illustration of rapid fluid mixing by magnetic beads on a rotating disk reported by Grumman *et al*.; reprinted with the permission from Royal Society of Chemistry [24]; (**b**) TOP: Time-lapse images of fluid mixing on a rotating disk by reciprocating centrifugal motion. (i) Liquid is added to the loading reservoir; (ii) high rpm during spinning results in compressed air in the pressure chamber; (iii) Decrease in spinning speed results in relaxation of air and pumping of liquid toward the center; (iv) spinning rpms are increased to the maximum level; (v) the siphon is primed as the liquid rises over the siphon crest; (vi) system is emptied; BOTTOM: Flow reciprocation work cycle profile. Reprinted with permission from the American Institute of Physics [30].

Researchers have also developed a method of mixing without magnetic beads. Pure fluids can be mixed by inducing “twisting” currents by rapidly switching the direction of the CD rotation. Grumman and coworkers were able to achieve similar mixing with this method and a pre-filled magnetic bead agitation method. The magnetic bead can be used in cases where the motor being used is less sophisticated and is not rated for rapid or frequent direction change. However, the change-in-direction method simplifies the system by removing the need for bead preparation and loading [24]. Another method for micro-mixing involving pneumatic pumping (discussed below in Section 2.4) has also been used in the rapid mixing of fluid [29]. In this case, repeated switching between centrifugal actuation and pneumatic pumping allowed for oscillation of fluids through a mixing chamber. Figure 1 displays some of the previous work with fluid mixing in CD centrifugal microfluidics.

### 2.2. Valving

Valving is a critical function in microfluidics and is usually passive in the case of CD applications. Most work has been done with either hydrophobic or capillary valves. Hydrophobic valves are used in cases where the width of the microchannel suddenly transitions from wide to narrow. Capillary valves operate when microchannels move from narrow to wide. A special type of valving which has recently been employed in mixing and metering applications is the use of the siphon valve. These valves are characterized by a siphon crest or area of the microchannel that changes direction at an area of the CD closer to the center than the level of fluid being transported. The key to siphoning is that the siphon must be primed by filling the microchannel with fluid. Once the siphon is primed, fluid can be passed through to the collection channel [31].

**Figure 2:**
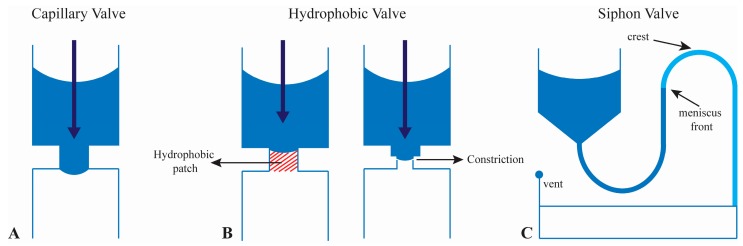
(**A**) Schematic of a capillary valve; (**B**) Schematic of hydrophobic valves using a hydrophobic patch or abrupt change in geometry; (**C**) Schematic showing a siphon valve where the siphon crest is located closer to the center of the CD than the meniscus front in the siphon microchannel. See text for details on the principles governing their function. Figure reprinted with permission of Royal Society of Chemistry [21].

The capillary valve is the most commonly used in microfluidic CD platforms (Figure 2A). In these valves centrifugal forces are manipulated to exceed or fall under surface tension forces to actuate or stop fluid, respectively [32]. These valves are often included on CDs with hydrophilic channel surfaces which allow fluid to flow passively along consistently sized channels. Spinning the discs propels the fluid down these channels, but when the channel abruptly widens the spinning speed (centrifugal force) must be increased to overcome the increasing surface tension. If the speed is increased over the “burst frequency” of the capillary valve then the valve opens and fluid is passed through. This technique allows for fluid flow to be precisely controlled by changing microchannel width and spinning speed of the CD. Chen and colleagues analytically explored the pressure barrier in a capillary valve. It was discovered that the burst frequency, or speed at which the valve opens, is increased as channel width is decreased, aspect ratio (depth:width) is increased, and the angle of channel expansion is increased [33].

Hydrophobic valves are another common valve type and are accomplished in one of two ways, as shown in Figure 2B. The first is through changes in channel geometry, by abruptly changing channel width from wide to narrow [34]. The second utilizes surface modification to induce regions of hydrophobicity, usually on hydrophilic substrates [35]. One way this can be done is to deposit hydrophobic polymer material onto a hydrophilic channel substrate. The regions of differing surface chemistry create a hydrophobic valve [36].

Siphon valves, as previously mentioned, are another variation of passive valves used in CD microfluidics [19,20] (Figure 2C). Using hydrophilic channel treatments or materials allows for the flow of fluid through the siphon valve. The initial flowing of fluid through the valve is termed priming and allows for continuous flow of fluid through the valve despite the physical location of the siphon crest [37]. Typically, siphon valves have an input reservoir connected to a siphon channel. This channel extends toward the center of the CD, opposing the centrifugal force. Once primed, the siphon channel is able to actuate fluid toward a collection chamber. During high-speed rotation, centrifugal forces are greater that the capillary forces restricting the fluid from overcoming the siphon crest. But when speeds are lowered to a critical speed, the capillary forces overcome the centrifugal force and actuate the fluid through the valve and toward the collection reservoir. Hydrophilicity is the main drawback of siphoning on the CD. Most often, this is solved by surface treatment of an originally hydrophobic surface. Surface treatments complicate fluidic behavior, as they can change or degrade over time, causing is a reduction in surface energy [38,39].

There are also a small number of active valving methods. Active valving is more robust than passive valve applications but there is an additional requirement of an external trigger. One such example is the infrared (IR) irradiated wax plug technology by Abi-Samra *et al.* [40]. This work centers on using an IR heat source to melt wax plugs inserted within the microchannel during spinning of the CD. Waxes with multiple melting points were used to perform complex, multi-stage assays on the discs. Another example is the work of Haeberle and coworkers, which relies on permanent magnets embedded and fixed on the CD surface. The permanent magnets on the CD create an oscillating magnetic field by passing over a set of fix magnets in the platform below. The displacement of the magnets on the CD actuates valves that allow air into the channels. This air is then used to direct fluid across the chip [41].

While valving is a key component to almost all CD microfluidics applications, quantification of specific parameters (*i.e*., rotational speed, spin duration, acceleration, *etc*.) are difficult to generalize. Each fluid actuation parameter is dependent on the particular application, channel geometry, and sample of interest. Therefore spin regimens for each valve type are most often specific to a particular application. For example, Siegrist and colleagues demonstrated fluid pumping on a microfluidic CD through a series of siphon valves using a low-to-high alternating rotational speed of 600 to 1000 rpm respectively. The speed at which the siphons were primed in this case was 770 ± 40 rpm. In this case, the siphons themselves were 100 µm deep and 1 mm wide [31]. In a later work, Siegrist and coworkers then used siphon pumping for sample lysis validation with a spin speed of 2000 rpm and a priming speed of 150 rpm with channels 200–300 µm deep [34]. Similarly, in a demonstration of pneumatic pumping as a method for siphon priming, Gorkin and colleagues recorded siphon valves being primed at ~1000 rpm after air was compressed in a partially filled chamber by spinning at speeds up to 7000 rpm. These microchannels were also 100 µm deep [42]. The same variability can be seen in works focusing on capillary or hydrophobic valves. For researchers to best understand the spinning parameters suited for their work, specific application and microchannel geometry should be considered, along with results from their own preliminary studies.

### 2.3. Volume Definition (Metering)

**Figure 3:**
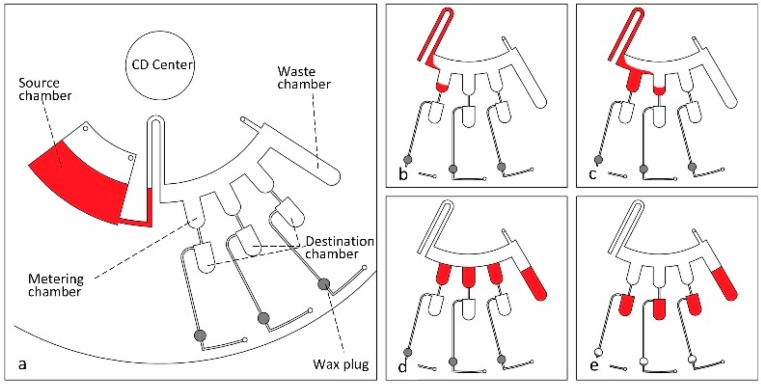
(**a**) All disc components labeled and fluid is loaded into the source chamber; (**b**) Rotation speed is increased so that the siphon valve is primed and the first metering chamber is filled; (**c**) Second metering chamber is filled; (**d**) All metering chambers are filled and the rotation speed is increased to move excess fluid to the waste chamber; (**e**) Rotation speed is increased again to open the hydrophobic valves and the wax plugs are melted to release trapped air in the destination chambers, creating a vacuum which allows fluid into destination chambers. Reprinted with the permission of PLOS [43].

Volume metering, or the precise measurement and movement of pre-defined volumes of fluid (µL–nL range), is a critical component to the translation of CD microfluidic technologies [19]. The ability to measure and then move finite volumes makes many assays possible that would otherwise require precise pipetting or tedious mixing methods highly susceptible to human error. In CD platforms, metering if most often accomplished through manipulating hydrophobic or capillary valving strategies and motor frequency to stop or move fluid. Other techniques, such as the induction of air compression or vacuum within the CD, are also used to move fluid in fixed volumes. An example of this metering is demonstrated in the Figure 3 below. In this case, motor speed and capillary valves within a hydrophilic channel are used to move fluid into position. Then, wax plugs are melted to induce vacuum or compression which allows the fixed volume to move into the destination chambers. This process is presented as Vacuum/Compression Valving (VCV), and demonstrated via metering in the work of Al-Faqheri and coworkers [43].

### 2.4. Multidirectional Actuation

While there are many advantages to actuating fluid using centripetal force, several researchers have highlighted the limiting nature of the unidirectional flow of fluid in response to this force. Because fluid only travels from the center of the disc toward the outer edge, the available space on the CD is greatly decreased. This limitation increases the difficulty of conducting complex, multi-stage mixing or detection applications. In order to maximize space for more intricate designs, researchers have looked at using methods to pump fluid back toward the center of the CD by capitalizing on the pressure created inside microchannels during centrifugation. These areas of pressurized air within the CD can be manipulated actively (with additional actuator) or passively (without additional actuator) to move fluid back toward the center of the disc [42].

Passive strategies for actuating fluid back toward the center of the CD include manipulating capillary action [20,37] and pneumatic pumping [42]. Capillary action is modulated by treating hydrophobic CD materials with hydrophilic surface treatments to increase surface energy. However, these treatments are not permanent and their degradation over time leads to changes in fluid behavior [38]. Pneumatic pumping does not require additional actuators or surface treatments. This technique aims to use the compression of air in a sub-compartment to actuate fluid by changing the frequency of CD spinning. At lower speeds the trapped air relaxes and expands, thereby forcing the fluid through the desired microchannel network. Gorkin and colleagues demonstrated the viability of this technique by priming a siphon microchannel without the use of hydrophilic treatments or hydrostatically forcing fluid over the siphon crest [42].

### 2.5. Fluid Actuation: Challenges, and Recommendations for the Extreme POC

Actuation of fluid by centrifugal force is at the core of CD microfluidics at the extreme POC. This method of actuation fits well in these applications because of the lack of need for extraneous equipment, power supplies, and the ability to perform assays on a singular disc. For every application concerning actuation of fluid using centripetal force (mixing of fluid, valving, metering, multidirectional flow, *etc*.) there are active and passive methodologies. For the purposes of the extreme POC, passive methods offer the more favorable experimental conditions. Passive applications do not require external components for actuation or additional embedded mixing particles, however, these applications may require a more robust motor for spinning the disc. This degree to which the motor can change direction or accelerate/decelerate is directly proportional to the ability to passively actuate fluid through an entire assay process. The increase in motor requirements could become a cost-limiting factor in some extreme POC applications.

Actuation of a CD microfluidic system for the extreme POC should focus on a passive mixing method, which employs a change-in-direction method where turbulent flows can be induced to quickly increase the diffusion rate of different fluids. Valving should also be accomplished through passive means, with combinations of hydrophobic and capillary valves being employed. Material for the disc should be hydrophilic in nature to increase capillary action, with hydrophobic valving accomplished through abruptly changing channel width. Siphon valves increase the complexity of disk manufacture but also increase mixing and valving functionality. The addition of these valves should be measured against the motor control that required to efficiently prime and use them. Similarly, volume metering should be accomplished through a combination of hydrophobic and capillary valves. The pre-defined volumes metered should provide enough working room of the CD for subsequent mixing applications. Pneumatic pumping is a promising passive strategy for moving fluid back toward the center of the CD. This technique should be included to increase the functional area on the CD. The combination of these passive designs will require significant precision in manufacturing and design. However, the advantage of removing any external power supplies, extraneous mixing elements, or sample loading equipment outweighs this requirement.

## 3. Drive Mechanisms

One of the principle components of any centrifugal microfluidic platform is the drive mechanism, also referred to as the “spin stand”. Fortunately, the compact-disk market has been well developed by industry and there are many options for researchers seeking to use spin schemes to accomplish complex microfluidic tasks. In general, CD drive mechanisms can be categorized into three groups: *significantly complex* drive-analysis combinations, *moderately complex* programmable direct-current (DC) motor systems, and *simple* battery/manually powered systems. Table 1 below provides several examples of the current state-of-the-art in each category. However, with the specific focus of this work, it is important to note that there are a number of considerations that prevent many of these current mechanisms from being successfully translated to the extreme POC as detailed next.

### Drive Mechanisms: Challenges, and Recommendations for the Extreme POC

**Figure 4:**
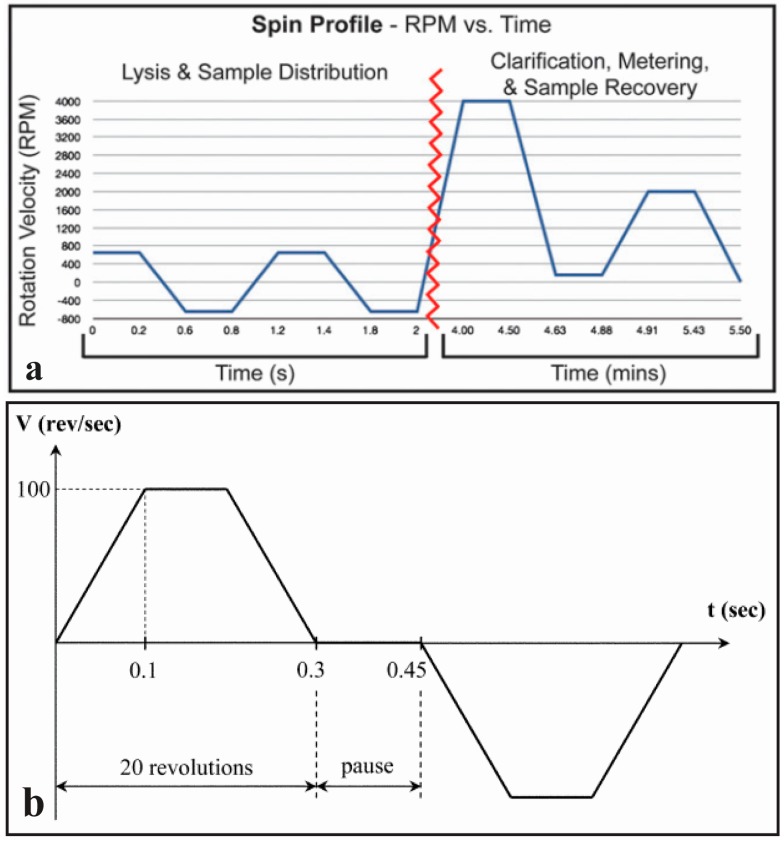
(**a**) Spin profile detailing motor velocity during lysis, measuring and recovering of the sample. Reprinted with permission of the Royal Society of Chemistry [34]; (**b**) Spin profile of cell lysis using rapid spinning in opposite directions to induce particle collisions. Reprinted with the permission of the Royal Society of Chemistry [55].

In the resource-limited environments presented in the extreme POC, one of the major concerns for resolving healthcare challenges is the lack of reliable electricity. The inaccessibility of a stable power grid make most of the technologies in the “Significantly Complex” and “Moderately Complex” categories in Table 1 obsolete. The majority of these systems are designed to convert a stable 120 VAC to a regulated 12 VDC for the power of the spin motor. Drivers paired with various DC motors for “moderately complex” systems require the conversion of the DC voltage into a pulse-width-modulated (PWM) signal controlled by the programming software. Even if the motor is powered by an independent 12 VDC source, such as an automobile battery (or similar size battery), the fact that a PC-based control interface is still required is a challenge for the extreme POC. Another limiting factor may be the complexity of the programmable spin protocols themselves. In the case of many of the complex assays accomplished on centrifugal microfluidics to date, multi-stage approaches with sequential spin speeds and durations have been used as illustrated in Figure 4. These steps must be optimized depending on the nature of the sample and the assay to be implemented. Although, it is envisioned that for an extreme POC application this optimization will take place in a well-equipped laboratory developing the diagnostic assay, the challenge still remains to enable these complex protocols in a platform amenable for extreme POC. Hence, a significant challenge is to equilibrate the need for these complex spinning protocols and the complexity of the platforms that can be implemented in practice. Further research into the nature of valving and fluid manipulation in disc-based devices will prove beneficial in this area to simplify the required spinning protocol.

Regarding the drive mechanism, the authors recommend that researchers focused on the extreme POC develop CD microfluidic techniques amenable to the ”Simple” CD drive mechanisms described in Table 1, or implement inexpensive and reliable power sources to allow for “Moderately complex” CD drives. Developing microfluidic platforms to operate on battery or manually driven motors will require design considerations minimizing multi-stage spin protocols, minimizing bi-directional spinning requirements, and balancing CD capacity with motor torque, speed and power requirements. The required torque is important and will depend not only on the inherent weight of the device, but more importantly on the amount of sample to be analyzed. While simple, battery powered devices may allow for increased functionality, CDs that can be manually driven or charged would be the ideal case. Special considerations should be taken in this case to engineer variability-reducing measures that would allow for repeatable results across a broad spectrum of operators, environments, or applications.

## 4. Manufacturing

**Figure 5:**
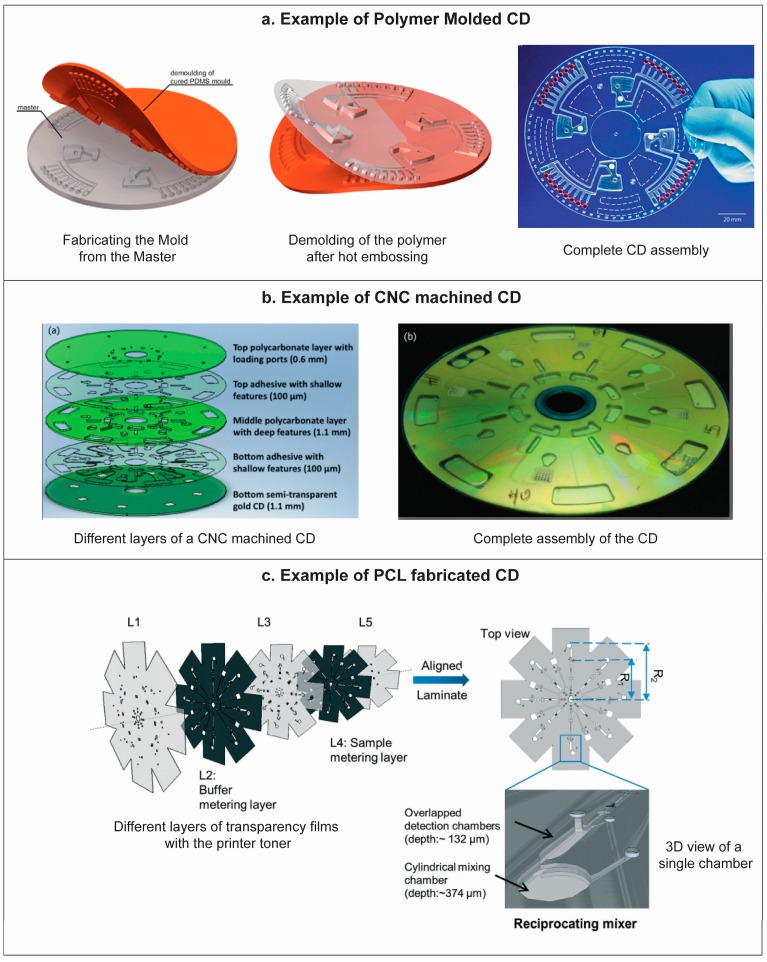
(**a**) A typical example of polymer molding of CD, where the CD device is fabricated by hot embossing of a polymer using a PDMS mold which is prepared using a CNC machined PMMA master. Reprinted with permission from the Royal Society of Chemistry [60]; (**b**) Schematic of the different layers of a CNC manufactured CD microfluidics device comprised of three polycarbonate and two PSA layers (left) and photograph of the assembled CD device (right). Reprinted with permission from the American Institute of Physics [30]; (**c**) Schematic of different layers of transparency films with printer toner and the assembled CD device with a 3D view of a single chamber, fabricated with PCL method. Reprinted with permission from the Royal Society of Chemistry [59].

We can categorize the fabrication methods of centrifugal microfluidics disk into two broad divisions: Polymer molding and computerized numerically controlled (CNC) machining [56]. Polymer molding can be used for low cost mass fabrication of CD devices. The master mold required in this case is a negative replica of the desired CD design. Different kinds of mold inserts can be created with photoresists, silicon, plastic and metal, and different methods like photolithography, deep reacting ion etching (DRIE) and micromachining can be applied to make these mold-inserts. Once the mold is created, different molding processes e.g., casting, hot embossing and injection molding can be employed for the mass production of the plastic discs. An illustration of one case of polymer molding of a CD device is presented in Figure 5a. Polydimethylsiloxane (PDMS), polycarbonate (PC), optical quality polycarbonate (OQPC), polyurethane (PU), polystyrene (PS), polyvinyl butyral (PVB), polymethyl methacrylate (PMMA) and cyclic olefin copolymer (COC) have been used as disc material in this approach [56]. For example, in the case of PDMS the master mold has been fabricated from SU-8, a negative photoresist, following soft lithography. Hot embossing techniques can be employed to fabricate PC, OQPC, PMMA, PS and COC disks. PMMA and OQPC were used to make the disk by injection molding technique. 

In CNC machining, different fluidic channels and chambers are machined in plastics such as PC, PMMA or COC. This technique generally uses multiple machined plastic layers that are later aligned to each other and bonded using patterned pressure sensitive adhesive (PSA) [57], tape or liquid adhesives. Figure 5b shows a typical centrifugal microfluidics disk using plastic and PSA.

A recent approach includes the stacking of patterned plastic films which are bonded using inkjet printers [58,59]. In this method, pre-designed patterns are printed on transparency films with patches of toners by using a laser printer followed by laser ablation of the architectural features of the CD *i.e.*, reservoirs and holes. After printing and laser ablation, the transparency films are aligned and roll pressed with a heated roll laminator. During lamination, the toner on the transparency films is melted and bonded with neighbor film. As printing, ablation and lamination are the three main step in this approach, it is termed as print-cut-lamination (PCL) method. In Figure 5c, different layers of the transparency films with printer toner are shown along with the 3D view of as prepared revoir on the CD device.

### Manufacturing: Challenges and Recommendations for the Extreme POC

To meet some of the World Health Organization, Special Programme for Research and Training in Tropical Disease (WHO/TDR) ASSURED criteria the CD device should be affordable and robust. In terms of robustness, present manufacturing techniques, whether polymer molding, CNC based manufacturing, or the PCL method, all provide suitable maintenance of centrifugal microfluidics device integrity during handling and assembly. This is helpful for transportation to remote areas and for usage in extreme rural environments. The affordability criterion includes the manufacturing cost as a strong determinant of the overall cost of the device. Polymer molding is a suitable technique for low cost mass production only when the number of devices to be made justifies the cost of a typically expensive master mold. The need for several thousand devices would justify this technique. CNC-based techniques and the PCL technique can be quite straight forward. These fabrication processes are serial and the price per unit can actually be cheaper than polymer molding for hundreds of devices. PCL can even have a slight advantage over CNC since the materials needed, films vs. plates, would likely be cheaper. Affordable CNC-based machines and laser-based plastic ablators can be comparable in initial price but the fact that CNC requires a number of different tools, depending on the complexity and dimension of the cut, can make laser engraving a preferable choice. Laser ablation also tends to be significantly faster than machining. However, CNC may be preferred over laser ablation if cut precision is important. If the lateral dimensions of the device are above 200 μm, the integration of xurography with the PCL technique may lead to inexpensive devices [57]. Laser ablation in the PCL method can be replaced by xurography by implementing an inexpensive and simple cutting plotter machine. The xurography method is preferable for structural and adhesive films, soft polymers with thickness below 1 mm. Table 2 below documents functionality of these manufacturing techniques as highlighted in the literature.

## 5. Assays and Reagent Compatibility

One of the major advantages of spinning CD microfluidic designs is that actuation is independent of physiochemical fluid properties (*i.e.*, pH, ionic strength, or chemical composition). For this reason, many types of fluids have been processed using CD microfluidics. These include whole blood, blood plasma, mucus, urine, and milk [19]. The versatility of platforms regarding sample fluid type has led to many filtering, sorting, mixing, and immunoassay combinations [15]. For the purposes of the extreme POC, the fluids and reagents discussed here can be categorized into one the following: biological fluids or assay reagents.

### 5.1. Biological Fluids

Whole blood, saliva, milk, and urine have been the primary biological fluid targets for CD microfluidic applications. These fluids have been targeted due to the presence of genetic information, environmental, or metabolic byproducts. These biological fluids have been analyzed to detect the presence of pathogenic cells (*i.e.*, bacteria, viruses, cancerous cells) or to measure expression levels of metabolites in response to environmental factors. Park and colleagues employed CD microfluidics and a combination of valving schemes to isolate circulating tumor cells (CTCs) from whole blood. Blood components were sequentially separated through opening and closing a series of capillary valves and manipulating disc rotational speed. CTCs were then bound to microbeads through a final mixing step which allowed for subsequent analysis [73]. Duffy and colleagues pumped urine through a microfluidic network equipped with a series of passive valves. Flow rates ranging from 20 to 6 μL/s were accomplished on the CD platforms [74]. However, one of the issues with analyzing biological samples on centrifugal discs has been the need for a series of complex mixing and washing steps. Schaff and Sommer addressed this issue by pursuing a method to analyze whole blood samples without these complex steps by combining cells with labeling antibodies and capture micro-beads in a single step. The cell pellet present at the periphery of the disc after spinning was laden with detection properties which could then be analyzed to quantify the amount of target analyte present [75].

An important consideration is how the nature of the sample changes depending on the application being targeted. A number of diagnostics can be implemented using just a few microliters of sample, since the targeted species are in high concentration. Assays that target a molecule fall into this low sample volume category due to the specificity of the molecules present for a given condition. Other applications require up to few milliliters of sample given that the targeted species are highly diluted. The detection of a pathogen causing infection is a good example. Hence, the volume to be analyzed in the extreme POC will range from few microliters to several milliliters.

### 5.2. Assay Reagents

#### 5.2.1. ELISA Reagents

**Figure 6:**
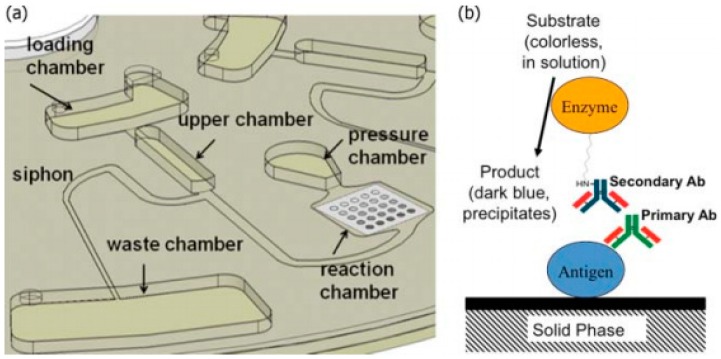
(**a**) Schematic of fluidic system composed of a series of hydrophobic and capillary valves. The siphon valve, along with a reciprocating spin protocol, was used to expose the sample to the reaction chamber for detection; (**b**) Schematic of antibody capture at each array element in the reaction chamber. Reprinted with permission from the American Institute of Physics [30].

One of the primary uses of CD microfluidic technology is the development of rapid enzyme-linked immunosorbent assay (ELISA) platforms for the testing of antibody and other protein concentration in the blood. ELISA works by detecting an immobilized antigen of interest through binding of a specific enzyme-conjugated antibody. This enzyme is capable of producing a detectable colorimetric signal when reacted with its substrate. By measuring the enzymatic signal produced, the presence of antigens or antibodies may be relatively quantified, allowing for the detection of certain diseases (*i.e.*, HIV, West Nile Virus) [76]. Several researchers have sought to develop this technique on a CD, which would revolutionize infectious disease testing in developing countries by reducing assay time and removing some of the tedious, error-prone nature of the conventional protocol. Lai *et al.,* demonstrated that centrifugal and capillary forces could be manipulated on a CD with optimized burst valve location and microchannel specifications to achieve a comparable detection range for ELISA with respect to the conventional 96-well microtiter method [77]. This study also revealed that ELISA on a CD could lead to reduced reagent consumption which, when combined with reduced assay time, could prove valuable in the extreme POC environment. The reagents utilized in these experiments consist of buffer solutions for sample washing, a blocking protein solution to restrict non-specific binding, and antibody solutions for detection. These materials must be kept isolated until the time of mixing, especially antibody solutions. The detection phase of these assays must also be highly regulated by valve schemes and channel dimensions to successfully separate amplified fluorescent sample from waste material. Noroozi and coworkers developed a semi-automated immunoassay based on a complex reciprocating spin regimen and fluid actuation through a siphon valve. Figure 6 below illustrates this work. Other researchers, such as Lee and colleagues have developed fully automated platforms with assay times less than 30 min with similar detection ranges. However these systems have utilized laser irradiated Ferrowax micro-valves (LIFM), therefore requiring equipment that would be difficult to implement in the extreme POC [72].

#### 5.2.2. RT-PCR Reagents

**Figure 7:**
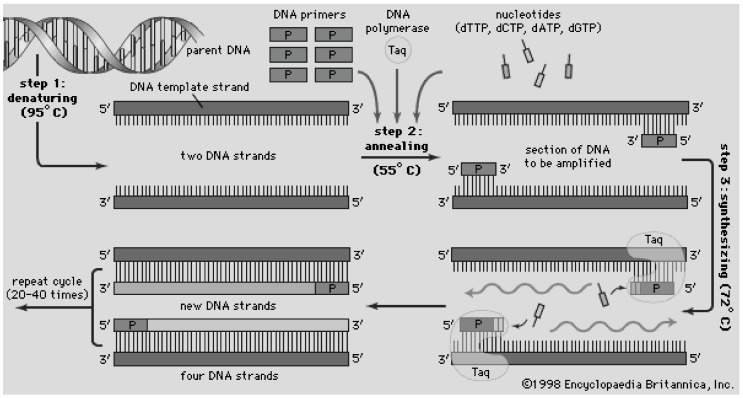
Schematic of the polymerase chain reaction, where a DNA sample is combined with DNA polymerase (Taq as in sample A of bottom image) and exposed to a thermal cycling profile which denatures, anneals DNA with a designated primer, and synthesis (amplification) of new DNA strands. Accessed online [81]

Real-time polymerase chain reaction (RT-PCR) is an assay characterized by combining nucleic acids (*i.e*., DNA or RNA) with the polymerase enzyme and short, highly-specific, amino acid sequences called primers. This combination leads to the replication of certain target proteins which can then be quantified to determine a relative level of expression within the original sample. Figure 7 illustrates the principles of this technique. Because this assay focuses on analyzing genetic information, there are several key applications of this technology at the extreme POC, including congenital disease detection. The reagents associated with the entire RT-PCR process can be separated into two phases. The first phase, DNA isolation and purification, requires a lysing agent (*i.e*., Trizol^®^ (Life Technologies, Carlsbad, CA, USA) in conventional applications) and a series of reverse transcription agents converting RNA to cDNA. These reverse transcription agents are often acquired in a kit containing buffer solution, random primers, reverse transcriptase enzyme, RNase inhibitor, and deoxynucleotide triphosphates (dNTPs). All of these components would be needed, along with the ability to catalyze the reaction of these components by cycling through a specified temperature profile, ranging from 4 to 85 °C. The second phase, polymerase chain reaction, requires the combination of pre-designed primers, polymerase enzyme with fluorescent tag, and buffer solutions. Cycling through a thermal program is again needed to replicate the cDNA of the gene of interest.

Due to the complex nature of the protocol and the large number of reagents and specific temperatures needed, researchers have attempted to accomplish either the first or second phase on the CD platform. Cho *et al.,* developed a method for extracting DNA from whole blood on a CD by using LIFMs and magnetic particles conjugated with specific antibodies. Results showed that pathogen-specific DNA was able to be isolated in less than 12 min, with subsequent PCR results being comparable to the conventional bench-top protocol [78]. Lutz and coworkers used a foil-based CD cartridge and a specialized centrifugal analyzer capable of 37 °C incubation to successfully perform phase two of the RT-PCR process [79]. The work of Amasia and colleagues aimed to greatly reduce the time of the amplification (second) phase of the PCR process by integrating a heat and ice valve system to recreate the effect of a thermocycler [80].

#### 5.2.3. Protein Assays

Biochemical assays focusing on protein and glucose concentrations have also been demonstrated on CD platforms. These assays require the storage of separate biochemical standards that can be exposed to colorimetric enzymatic reagents and then compared with the biological sample. This comparison allows for qualitative diagnoses to be formed based on the known concentrations of the standards. La and coworkers utilized the precise metering and mixing capabilities of CD microfluidics to detect the concentration of glucose present in specified samples [82]. The path to the extreme POC is more clear for these type of assays as the number of steps required for single-end-point assays being compared to pre-determined standards has a much smaller sample preparation and detection barrier than the previously discussed procedures. Rothert and colleagues demonstrated asimplified chip design of such a CD, with easily separated sample and reagent chambers and a well-defined mixing chamber where *Escherichia coli* cells were used to fluorescently (GFPuv) quantify arsenite and antimonite analytes via the ArsR protein [83].

### 5.3. Assays and Reagent Compatibility: Challenges and Recommendation for the Extreme POC

One of the challenges for extreme POC applications for each of these applications and similarly complex biochemical assays will be sample loading, storage and stability. In each case discussed here, samples and reagents were carefully loaded into CD platforms in controlled laboratory environments with specialized equipment. This equipment or expertise may not be available at the extreme POC. This concern leads to the need for storage or loading simplification schemes that allow healthcare workers to avoid the error-prone sample preparation steps not covered in the cited works. Generally, protein-based components such as enzymes or antibodies are packaged and stored in such a way that biochemical stability is maintained. Samples are delivered in a soluble dried form which can then be re-suspended in a solvent such as phosphate-buffered saline (PBS) or deionized (DI) water. This protein-solution must then be stored at a temperature below the denaturalization temperature to avoid changes or degradation to the product. Often this storage temperature is around 4 °C, which exceeds the limitations of the general refrigeration that is scarcely available at the extreme POC.

Furthermore, stability, cross-contamination, and degradation become challenges when considering the necessary shelf life of extreme POC devices. Devices must be manufactured, shipped, received through custom patrols, distributed through poorly designed or maintained transport channels and then properly stored until use. All of these factors suggest that significant effort must be devoted to developing packaging that will isolate assay components from both the external environment and other components themselves. Such packaging would maximize shelf life and reduce the need for difficult and expensive transport of assay components and devices.

Potential solutions to this issue may be the incorporation of the more stable dried soluble component into the microfluidic chambers during the manufacturing process. In this way, solvents could be added or mixed with these samples immediately prior to conducting the test. However, further work must be done to develop robust assays and solutes capable of being suitably re-suspended in likely available solvents (*i.e*., minimally filtered or sterilized water, bottled water). Alternatively, more suitable solvents (*i.e*., PBS, DI water) may be included in isolated compartments along with the other components. Consideration should also be given to the increased cost associated with incorporating protein-based components into the CD manufacturing process.

Alternatives to protein or antibody-based approaches include the use of more robust labels such as enzyme-substrate reactions [84,85], aptamers [86,87], or nanoparticles conjugated with small detection molecules [88]. Both enzyme-substrate reactions and aptamers are viable alternatives because of their high specificity and ability for detection to be regenerated multiple times without loss of affinity of specificity [89]. Nanoparticle based approaches lose some of the selectivity of these other approaches but they offer the ability to capture a wide range of bio-particles with a single functionalized particle [89]. In each of these cases all reagents would need to be pre-loaded into the CD in a stable fashion prior to shipping, as the facilities needed to develop these products would not be available at the extreme POC.

The infrastructure that is currently needed to implement a number of the assays on the CD includes specialized equipment, such as thermocyclers, centrifugal readers, and lasers may be necessary to accomplish some of these techniques on CDs. This may limit the application of these technologies to the extreme POC, where cost and stable power supply are formidable limiting factors, even for a centralized health clinic. While PCR-based detection is an amenable technique in clinical diagnostics it is unlikely to become a solution for the extreme POC. Different detection mechanisms are detailed in the next section.

## 6. Detection

Detection is one of the major aspects of centrifugal microfluidics towards point of care applications. Though optical detection approaches are mostly adapted in compact disk microfluidics platforms, several other approaches, including electrochemical detection, have also been applied. An overview of different detection techniques is presented here with their working principles and selected applications.

### 6.1. Optical Detection

For detection, optical detection has been primarily used in centrifugal microfluidics platform for quantitative proteomic analysis and infectious disease diagnostics. Optoelectronic technology has made it easy to couple optical systems on to the CD platform for detection. Optical detection applied to CD microfluidics can be divided into three major categories: fluorescence detection, absorbance detection, and chemiluminescence detection.

#### 6.1.1. Fluorescence Detection

Among different optical detection methods, fluorescence detection is one of the most widely used sensing techniques for centrifugal microfluidics system because of its high sensitivity and selectivity. A fluorescent dye is a small molecule, protein or a quantum dot that emits a photon when excited with an appropriate wavelength. Different biomolecules, such as proteins, nucleic acids, or lipids, can be labelled with fluorescent dyes. In such cases, a required fluorescence detection system would feature at least three components: light to excite the fluorescent particles, fluorescent dyes to tag non-fluorescent particle and a detector to capture and record the emitted photons for further analysis. Researchers have adapted fluorescence detection on centrifugal microfluidics disk to analyze the performance of different immunoassays. For example, Puckett *et al*., used fluorescence detection to analyze the performance of homogeneous, protein-based assays on a centrifugal microfluidics platform [52]; Riegger and co-authors investigated color-multiplexed bead-based fluorescence immunoassays on a centrifugal microfluidics platform by using sets of beads that were functionalized with antibodies tagged with different quantum dots and dyes. These beads were collected in the detection chamber where they came into contact with a sample containing different analytes. As a proof of principle, they successfully identified hepatitis A and tetanus on microfluidic lab-on-a-disk platforms [90].

Another example is the work by Rothert *et al*., who used fluorescence detection while demonstrating a reporter-gene-based whole-cell biosensing system on a CD platform. They used a reporter protein as the sensing unit and measured the fluorescence intensity of the protein upon binding to the inducer on a CD platform. The fluorescence intensity measured from the protein is directly proportional to the concentration of the inducer. Using this technique they showed that the detection time in their system was significantly shortened. This could prove useful for detection of hazardous compounds and chemical/biological warfare agents as well as identification of compounds of biological and pharmacological interest [83].

#### 6.1.2. Absorbance Detection

Absorbance detection is based on the absorbance capability of the analyte while exposed to a light of certain wavelength. A beam of light loses energy when passing through a sample and this absorption depends on the atomic and molecular structure of the sample. In an experimental setup, sample absorbance depends on the optical path length of the holder and the sample concentration. Since the sample volume used in microscale experiments is small, the optical path length is also small and the absorbance strongly depends on the sample concentration. The requirement for high sample concentration and short optical path length are challenging for absorbance detection methods to be implemented in microfluidic applications [91], but a few researchers have succeeded in adding absorbance detection capabilities to a centrifugal microfluidics platform.

Lee *et al*., implemented an absorbance detection method in their portable and fully automated ELISA-based CD device to test infectious diseases from whole blood [72]. They first separated plasma from whole blood by centrifugation. The plasma was then mixed with a horseradish peroxide (HRP)-labeled detection antigen and antigen-functionalized polystyrene microbeads. In their detection chamber, absorbance at 450 and 630 nm wavelengths were measured by the optical detection module coupled with their portable analyzer. Once captured, the absorbance signal was amplified using a trans-impedance amplifier and later digitized to facilitate its reading and analysis. They demonstrated their technique for the detection of Anti-HBs and HBs-Ag.

**Figure 8:**
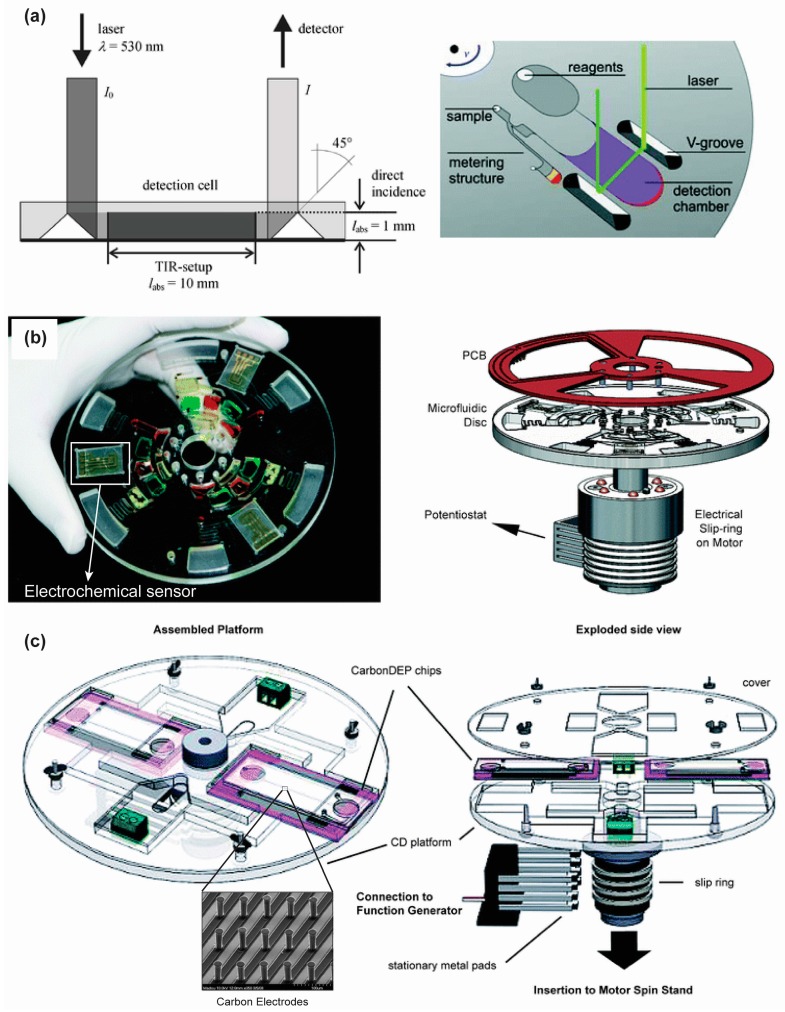
(**a**) Principle of absorbance detection adapted by Steigert and colleagues. Reprinted with permission from the Royal Society of Chemistry [92]; (**b**) Photograph of the CD device with the electrochemical sensor within it (left) and the schematic of the set up for electrochemical sensing. Reprinted with permission from the Royal Society of Chemistry [93]; (**c**) Experimental set up for 3D carbon electrode dielectrophoresis and schematic of the complete set up for electrical connection. Reprinted with permission from the Royal Society of Chemistry [94].

Steigert and his colleagues integrated absorbance detection in their micro-structured disposable polymer disk and employed that for direct measurement of hemoglobin [45] and alcohol concentration [92] in whole blood using a laser beam. In their polymer disk set up, triangular V grooves were monolithically embedded near the detection chamber. While upon incidence on the side facet of the V-groove, total internal reflection of the laser beam occurred. Consequently the beam was directed to travel through the detection chamber and again reflected at another V-groove towards a spectrophotometer as shown in Figure 8a. Since the beam intensity was attenuated according to the initial hemoglobin or alcohol concentration, the optical signal was recorded in the spectrophotometer. Direct data processing which was based on a measured calibration curve yielded to the requested result.

#### 6.1.3. Chemiluminescence Detection

When using a chemiluminescence (CL) detection method, one is interested on the binding of the analyte with the target which causes certain chemical reactions that result in a photochemical emission. Ducree and his group developed a method for conducting common chemiluminescent assays on CD through a parallel readout scheme [46]. They carried out an antigen-antibody reaction between antibody-functionalized polystyrene beads and the target antigen in the detection chamber of the CD device. The beads were then coupled with detection antibody labelled with HRP. Photochemical emission occurred in presence of HRP when Luminol/H_2_O_2_ was flowed over the beads. The number of photons emitted is proportional to the antigen concentration. The emitted photochemical signal was recorded by a PMT tube and analyzed with a previously calibrated curve. They demonstrated this technique for the detection of cardiac markers. The bead-based chemiluminescent ELISA procedure was able to capture antigens indicative of acute myocardial infarction (AMI) on their rotating polymer disk.

Date *et al*., employed chemiluminescence detection in their spore based sensing system on portable centrifugal microfluidics platform to measure the concentration of arsenic in water and also in blood serum [49]. They used *Bacillus subtilis* *ars*-23 as arsenic sensing bacteria spore. On a CD platform, the arsenite containing sample, bacteria spore suspension and a chemiluminescent substrate were transported to a detection chamber by centrifugation. Photon emission occurred due to photochemical reaction between the reagents and was recorded by a spectrophotometer. They achieved a lower detection limit of arsenite both in water and serum than that of other available techniques. The authors envisioned the use of such portable CD device for on-site arsenic detection in rural areas of developing countries.

### 6.2. Electrochemical Detection

The electrochemical detection unit on a CD-based centrifugal platform uses a three-electrode set up composed of a working electrode, a reference electrode and a counter electrode. The electrical environment around electrodes changes when antibodies or cells get adsorbed on the electrode surface. The change in the electrical environment can be measured by a choroamperometric plot or cyclic voltammogram. The electrochemical detection unit is integrated with the rotating platform either by slip ring-brush set up (Figure 8b) [93] or liquid mercury based slip ring [95]. Kim *et al*., performed flow enhanced electrochemical detection on a rotating disk using C-reactive protein (CRP) as a model antigen [93]. Using flow enhanced electrochemical detection, they achieved a five-fold limit of detection (LOD) improvement over stationary electrochemical measurement and 17-fold LOD enhancement over optical detection. Nwankire *et al*., successfully demonstrated the label free detection of ovarian cancer cells (SKOV3) from whole blood using electrochemical impedance spectroscopy on a fully integrated, automated lab-on-a-disk platform [71]. They extracted the cancer cells from a whole blood sample and collected them in an electrochemical detection chamber. The electrochemical impedance was measured upon binding of the cancer cells with the specific antibody in the detection chamber. Li *et al*., presented a lab-on-a-CD device for parallel whole blood analysis based on electrochemical detection. They used a nanoporous gold electrode modified with multiwalled carbon nanotubes to increase the electrode surface area for better immobilization. The CD device successfully determined the concentration of glucose, lactate and uric acid in whole blood within a few minutes [96].

### 6.3. Detection: Challenges and Recommendations for the Extreme POC

Optical detection serves as the pilot detection method in centrifugal microfluidics platform. However, each optical detection method has advantages and disadvantages. Fluorescence detection offers high sensitivity and high selectivity in the sensing system, but needs an off chip optical system to visualize and analyze the signals. This makes the whole system complex and bulky. In some cases the signal needs to be amplified, which requires additional instrumentation. The off chip instrumentation can be miniaturized to make the whole device portable, but miniaturization increases the manufacturing cost and eventually the overall cost of the device. In comparison to fluorescence detection, chemiluminescence offers a more simple approach and requires no complex instrumentation. This reduces the cost of the whole system significantly and enhances the portability of the analytical devices. However, for successful adaptation in a disposable and easy-to-use CD microfluidic device, the chemiluminescence detection system needs to have low-cost, sensitive photodetectors which are still not available. In absorbance detection, a major drawback in microfluidics applications is its poor sensitivity. The short optical path length in microfluidic application reduces the absorbance signal and thus affects the sensitivity. Despite poor sensitivity compared to fluorescence or chemiluminescence detection, simple instrumentation and the ability to multiplex make absorbance detection suitable for portable, economical, and easy-to-use CD devices. Compared to optical detection, electrochemical detection methods are inexpensive, robust, and portable and they require a smaller instrumental footprint. Also, electrochemical detection does not need optical grade CD material as required for optical detection. The response time in electrochemical detection is also faster than the optical sensors [97]. To date, only gold electrodes have been used for electrochemical detection on centrifugal platforms, which may be expensive for extreme POC applications. Inexpensive electrodes like screen printed carbon sensors could be also employed on compact disk set up, although the printing of microscale electrodes that are closely spaced can be challenging. Electrochemical sensing can also be integrated with electrokinetic phenomena, such as dielectrophoresis, used for sample preparation. In this case, targeted biological cells are trapped on an electrode surface using positive dielectrophoresis force and any changes on the impedance of the electrode is measured for detection of the targeted cells [98]. As dielectrophoresis depends on the dielectric properties of the cell, the dielectrophoretic impedance measurement approach can provide a label free detection method on a CD platform. This approach could be employed to concentrate rare cells or pathogens from a whole blood sample to facilitate their detection on a centrifugal device. To this end, Martinez-Duarte *et al*., have already demonstrated the implementation of dielectrophoresis on the centrifugal platform using arrays of 3D carbon electrodes (Figure 8c) [94].

In resource-poor rural settings, there is often a lack of essential technology and trained personnel to perform detection signal analysis and interpretation. Hence, it is extremely important to ensure that the result from the assay can be interpreted easily and conclusively by someone with minimal or no training at all. Binary or colorimetric read-out should be employed in diagnosis devices to remove any ambiguity and make it suitable for the extreme POC (and even for the clinical setting). If the cost for miniaturization of the off-chip instruments and sensitive photodetectors is minimized, fluorescence or chemiluminescence detection could be candidates for this purpose. However, integration of disposable screen printed electrochemical sensors on the compact disk platform are currently a better option for the lowest cost, disposable and sensitive unit for detection. 

## 7. Multiplexing or Multi-Directional Flow-Several Patients at Once, or One Full Assay on Disc per Patient

**Figure 9:**
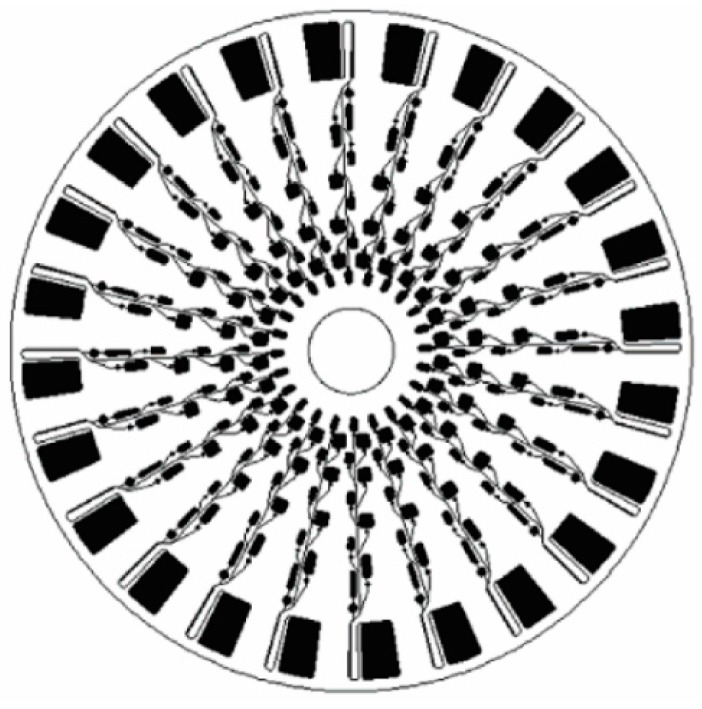
Schematic of CD-ELISA design with 24 sets of assays. Reprinted with permission from the American Chemical Society [77].

Multiplexing is the process of combining multiple processes on the same CD to be processed simultaneously. The ability to multiplex samples for multiple assays or for confirmation of a singular result is one of the advantages of CD microfluidics within the extreme POC. The economy of CD platforms is also greatly improved by being able to combine processes on one disc. To this end, researchers have multiplexed both multiple assays on a single disc and replicated a single assays multiple times. Work by Lai and colleagues is shown in Figure 9 and is focused on ELISA where 24 assays were placed on the same CD. This type of chip design maximizes the economy of this platform for use in the extreme POC as this allows for duplicates to be tested providing greater confidence in results, or multiple patient analyses in extremely resource-deprived areas [77].

### Multiplexing: Applications, Challenges and Recommendations for the Extreme POC

Multiplexing offers some significant advantages and disadvantages in terms of the extreme POC. The advantages of this technique include the ability to increase the economy of a singular chip by allowing multiple analyses of one or more samples simultaneously. This criterion alone could counterbalance an increased cost for more specialized equipment or reagent quantity. However, multiplexing must be balanced against the degree to which manufacturing complexity will increase based on CD design. Increased manufacturing complexity may eliminate certain material types or more cost-effective fabrication methods, thereby cancelling any added benefit of performing multiple tests on a single disk.

## 8. Disposal—An Important Consideration for the Extreme POC

One critical aspect of healthcare at the extreme POC which is not commonly considered in the development of CD microfluidic technologies is disposability of the platform after use. The inability to safely and efficiently dispose of a CD contaminated with blood or other biological samples could make an otherwise functional platform unusable do to the threat of environmental contamination. This topic has not been thoroughly discussed in the literature, however general degradation characteristics of the common materials (*i.e.*, PDMS, polystyrene, polycarbonate) suggest that these materials would degrade in soil over the course of several years, with this degradation being assisted by a warm, moist climate [99].

One strategy for disposability suggests the need for a material that is completely degradable through organic processes (*i.e.*, burning, hydrolysis). This strategy would require the breakdown of materials into organic degradation products that are naturally eliminated in the environment. Thermal degradation of PDMS was demonstrated by Radhakrishnan, where it was shown by thermogravimetric analysis to break down into a number of slowly degrading products, such as styrene [100]. Another strategy for disposability suggests that CDs may be reprocessed and reused through sustainable cleaning and repackaging processes. This strategy would require a separate set of material parameters which have yet to be thoroughly studied in the literature.

## 9. Conclusions

The above review of the literature points out many of the advantages that CD microfluidics could offer in applications at the extreme POC. Among these advantages are the ability to contain the whole assay in a single CD device without requiring external pumps and tubing for fluid actuation, a platform with reduced footprint, the ability to multiplex a variety of assays or samples on a single platform, the strict control of fluid through passive valving techniques, and the ability to pump biological fluids through microfluidic networks insensitive to the physiochemical factors that restrict other designs. However, this technology is not without its own challenges within the extreme POC context. The following list comprises the “Grand Challenges” of CD microfluidics at the extreme POC as seen by the authors:

1. Development of fluid actuation schemes, *i.e*., mixing and valving, that do not require the manufacturing of complex microfluidic channel designs and complex spinning protocols;
2. Development of technology to manufacture inexpensive, robust, and completely biodegradable discs for reliable transportation, storage, and easy and safe disposal of contaminated components;
3. Development of temperature-independent and robust reagents, and storage schemes to avoid contamination and degradation of assay components over long periods of time;
4. An assay result that is binary and can be unambiguously read by a broad audience regardless of their cultural or technical background;
5. Minimize the need for sample preparation, complex equipment, expertise, and controlled environmental conditions to decrease the likelihood of experimental errors and contamination.

These items present a specific challenge for engineers developing centrifugal microfluidic technology because they must all be balanced against maintaining a total cost that is affordable for the patients in the extreme POC, or their governments, but has a profit margin that is attractive enough to incentivize industry partners with the means and expertise to mass produce and distribute the technology. Discussions with non-profit organizations such as Medecins Sans Frontieres (MSF), the World Health Organization and the health ministries of the different governments are also of crucial importance. The current state-of-the-art depicts a challenging yet optimistic future for the application of CD microfluidic devices in the extreme POC. The upcoming solutions in this area will certainly need to compromise between platform capability and economy.

## Figures and Tables

**Table 1:** Summary of CD drive mechanisms.

Drive Description	Power	Control	Relative Cost	Special Capability	References
Significantly Complex					
Gyrolab Workstation™ (Gyros AB, Uppsala, Sweden)	120 VAC	Proprietary Software (Gyroloab Control (embedded), Gyrolab Evaluator (PC-based analysis))	$$$$	Laser-induced fluorescence detection, proprietary CDs, automated centrifugation control	[44]
Custom Colorimetric Hemoglobin Analyzer	120 VAC	Standard motor drive software, with custom coding for actuation and sample loading	$$$	Sample loading via Pipejet ™ (BioFluidix, Freiburg, Germany), laser and spectrophotometer, PC controlled actuation	[45]
Centrifuge with Photomultiplier Tube (PMT)	120 VAC	Embedded motor control software, PMT read-out software	$$$	PMT, *x*-*y* drive for PMT positioning	[46]
Moderately Complex					
CD-Read Only Memory (ROM) Drive	120 VAC	ASPI driver for PC	$$	Built-in laser driven optical system, limit of 6000 rpm	[20,47,48]
Stepper Motor + Driver	12 VDC for drive actuation 120 VAC likely required for driver and user interface (computer)	Programming language with development environment (*i.e*., Visual BASIC, C, C++, LabVIEW)	$$	Speed and torque only limited by rating of selected motor	[49,50]
Servo Motor + Driver		Programming language with development environment (*i.e.,* Visual BASIC, C, C++, LabVIEW)	$$	Speed and torque only limited by rating of selected motor	[42,51,52]
Simple					
Hand-powered Centrifuge for Anemia Diagnosis	Manual	Manual (training may be required for consistency)	$	Salad-spinner based design, approx. 600 rpm potential speed, tubes used (not CDs)	[53]
Egg-beater as Centrifuge for Blood Fractionization	Manual	Manual	$	Approx. 1200 rpm potential speed, tubing used (not CDs)	[54]

$–$$$$: Relative cost scale with “$” representing the least expensive options and “$$$$” representing the most expensive options.

**Table 2:** Summary of CD manufacturing processes. Only selected examples are reported.

Fabrication Technique	Material	Inexpensive	Disposable	Functionalities
Polymer Molding	COC	No	Yes	Extraction of plasma from whole blood [61], fully integrated metabolic assays on whole blood [62], direct measurement of hemoglobin from whole blood [45], hematocrit determination [47]
	PDMS	No	No	Purification of CD4+ cells from blood sample [63], identification and manipulation of single cell [64], blood separation [65], cell lysis [55], micro-assays [66]
CNC Machining	PMMA	Yes	Yes	Purification and separation of miRNA from whole blood [63], colorimetric analysis [67], liver function screening [68], purification of RNA [69], whole blood processing [70], label free cancer cell detection [71], immunoassay from whole blood [72]
	Polycarbonate	Yes	Yes	Cell lysis and nucleic acid extraction [34]
PCL Method	Polyester	Yes	Yes	Protein quantitation in blood [59]
